# Structured feature selection using coordinate descent optimization

**DOI:** 10.1186/s12859-016-0954-4

**Published:** 2016-04-08

**Authors:** Mohamed F. Ghalwash, Xi Hang Cao, Ivan Stojkovic, Zoran Obradovic

**Affiliations:** Center for Data Analytics and Biomedical Informatics, College of Science and Technology, Temple University, North 12th Street, Philadelphia, 19122 PA USA; Mathematics Department, Faculty of Science, Ain Shams University, Cairo, 11331 Egypt; Signals and Systems Department, School of Electrical Engineering, University of Belgrade, Belgrade, Serbia

**Keywords:** Structured feature selection, Block coordinate gradient descent, Gene expression, Microarray analysis, Prior knowledge

## Abstract

**Background:**

Existing feature selection methods typically do not consider prior knowledge in the form of structural relationships among features. In this study, the features are structured based on prior knowledge into groups. The problem addressed in this article is how to select one *representative* feature from each group such that the selected features are *jointly* discriminating the classes.

The problem is formulated as a binary constrained optimization and the combinatorial optimization is relaxed as a convex-concave problem, which is then transformed into a sequence of convex optimization problems so that the problem can be solved by any standard optimization algorithm. Moreover, a block coordinate gradient descent optimization algorithm is proposed for high dimensional feature selection, which in our experiments was four times faster than using a standard optimization algorithm.

**Results:**

In order to test the effectiveness of the proposed formulation, we used microarray analysis as a case study, where genes with similar expressions or similar molecular functions were grouped together. In particular, the proposed block coordinate gradient descent feature selection method is evaluated on five benchmark microarray gene expression datasets and evidence is provided that the proposed method gives more accurate results than the state-of-the-art gene selection methods. Out of 25 experiments, the proposed method achieved the highest average AUC in 13 experiments while the other methods achieved higher average AUC in no more than 6 experiments.

**Conclusion:**

A method is developed to select a feature from each group. When the features are grouped based on similarity in gene expression, we showed that the proposed algorithm is more accurate than state-of-the-art gene selection methods that are particularly developed to select highly discriminative and less redundant genes. In addition, the proposed method can exploit any grouping structure among features, while alternative methods are restricted to using similarity based grouping.

**Electronic supplementary material:**

The online version of this article (doi:10.1186/s12859-016-0954-4) contains supplementary material, which is available to authorized users.

## Background

The objective of supervised feature selection methods is to select a discriminative but concise list of features among a possibly large set of features in order to differentiate between classes. Using only a small set of features improves the accuracy and increases the interpretability of the classification model [1–3]. Several types of feature selection methods have been developed to address that problem. Filter-type methods select features independently from a classification model, whereas wrapper and embedded methods use feature selection as a part of training the classifier, which typically involves fitting more hyper parameters, and requires to use nested cross validations [4]. Therefore, wrapper and embedded types typically suffer from increased computational cost and possible overfit, especially when a small number of examples are available. Nevertheless, filter-type methods are allowed to utilize the labels of the subjects. The outcome of a filter-type method is the selected features list, regardless of their weights, where the selected features can be used later to learn a classifier. In this paper we focus on the filter type feature selection method.

In general, feature selection methods do not consider the structure among the features. For example, the features may be clustered such that the features in the same cluster are more similar to each other than features in different clusters. In many applications, the requirement is to select one feature from each group such that all features are jointly discriminative. This problem exists in many applications (see Additional file 1 for more details). Analytics of sports: One major objective of analytics in sports is to enhance team performance by selecting the best possible players and make the best possible decisions on the field or court [5]. Imagine that a coach needs to select a set of best players for the team. Intuitively, the set of all possible players can be grouped (based on their positions in the field) into *G* groups where each group contains all players who play in that position. Since the objective is to select the best team, one may claim that the problem can be solved by selecting the best player in each position separately. However, using this approach synergy among the players is not considered. For example, players 1 and 2 might be the best players for positions A and B, respectively, but the players might not be so cooperative as to be in the same team. Therefore, the idea is to select one player from each group such that the selected team has the best performance. Multivariate time series classification: This problem can be addressed by using discriminative multivariate temporal patterns that are extracted from each class [6, 7]. One example of such interpretable multivariate pattern is that if gene X and gene Y are up-regulated at the same time followed by the down-regulation of gene Z, then the patient is developing the condition. In order to discover such patterns, one can extract all patterns from gene X as one group and all patterns from gene Y as another group, and so on. In other words, the grouping structure among genes is based on all patterns extracted from one variable (gene). Therefore, the problem is to select one pattern from each gene. The list can be analyzed by another method to extract a low dimensional multivariate pattern. Dummy variables: Dummy variable is an artificial variable created to represent a categorical variable. Therefore, the coefficients of the dummy variables are naturally partitioned into groups, where it is naturally to select only one variable from each group. Microarray analysis: The genes can be grouped based on their correlation or similarity, based on prior knowledge about their molecular functions, a cellular pathway, or based on annotation by a specific term of the gene ontology [8]. Therefore, it would be enough to choose only one gene from each group.

The main advantage of performing analysis on groups of features is the compactness and improved interpretability of analysis results due to the smaller number of groups and greater prior knowledge available to such groups. In this study, we address a *novel* problem where the objective is to select a *representative* feature from each group such that the selected features are *jointly* discriminative. Our contribution can be summarized as follows. (1) We formulate the feature selection problem in order to select a representative feature from each group *simultaneously and jointly* as convex-concave optimization, which is transformed into a sequence of convex optimization problems that can be solved using any standard efficient optimization algorithm; (2) We develop a block coordinate gradient descent (BCGD) algorithm that is four times faster than any standard optimization algorithm for the proposed feature selection method; (3) The experimental results show evidence of the efficiency and scalability of the proposed algorithm. In order to evaluate the proposed method, we applied it to perform a feature selection for microarray analysis as a case study. Related work in feature selection for microarray analysis: Feature selection for microarray analysis has been extensively studied [9–12], where many of them can be categorized as filter based approach, in which genes are selected prior to learning the classification model. Attempts to address similar problems include clustering genes by utilizing the biological relevance of those genes and then using the representative medoid from each biologically enriched cluster *separately* [13, 14]. This clearly leads to a sub-optimal solution because it does not consider the interaction among genes from different clusters. This problem is addressed by proposing an efficient double sparsity optimization formulation that simultaneously identifies mutually exclusive feature groups from the low-level features (genes), such that the groups contain correlated features, and then the groups are generalized to higher level features [15]. The high-level features (metagenes) are constructed as a linear combination of low-level genes from that group. The problem with that method is that the meta genes might not be quite interpretable [16].

A Maximum Relevance Minimum Redundancy (mRMR) method was developed for feature selection of microarray data [17]. The method is based on mutual information criteria that maximizes the relevance of the feature to the target and simultaneously minimizes the redundancy to other features. The features are then ranked based on that criteria such that the high-rank features are the more informative features. Another method is proposed to select the most informative features while minimizing the redundancy among the selected features [18].

The problem is formulated as a quadratic programming formulation, which can be solved by any standard efficient optimization algorithm. However, the formulation involves a matrix that is not positive semi-definite; hence, it might lead to a poor local optima. A very recent method [19] formulates the problem as a convex formulation with two terms, one to select features with maximum class separation and the other to select non-redundant features. The redundancy among features is computed based on Pearson correlation, which is encoded as a positive semi-definite matrix. In order to apply the method for high dimensional data, the authors have applied low rank approximation to the quadratic term so that the solution can be found efficiently. Although those studies [17–19] look similar to our proposed method, their methods were developed particularly to minimize the redundancy among features, whereas our method is general enough to exploit any structure among features. In other words, the features can be grouped based on similarity such as Pearson correlation as in [19] or mutual information as in [17, 18], or based on any other prior knowledge about genes, such as molecular function. Therefore, our work can be exploited to any application where the features can be grouped in advance using prior knowledge.

The importance of selecting features from gene subsets or groups was recently studied [20]. The method first partitions the features into smaller blocks. Then, in each block, a small subset of *r* features are chosen based on their classification accuracy. Once the top-r features from each block is obtained, they are mutually compared to obtain the best feature subset. We note that the interaction among features are not fully considered but only the interaction among the top-r features from each subset. In addition, their method was developed using a wrapper-based approach, while the main focus of this paper is based on a filter-type feature selection approach.

## Methods

### Problem definition

**Fig. 1:**
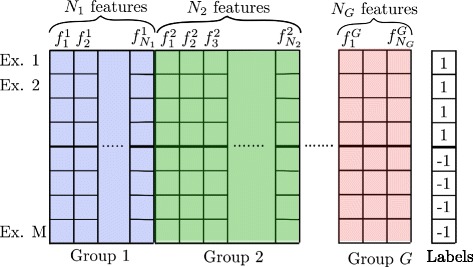
Matrix representation for the feature selection problem. Features are structured into *G* groups, where group *g* has *N*_*g*_ features, where *g*∈{1,2,…,*G*}. ${f_{i}^{g}}$ is the *i* ^*th*^ feature in group *g*

Let us assume that we have a dataset *D* of *M* examples (samples) and *N* features, where the features are structured into *G* groups such that the number of features within group *g*∈{1,2,…,*G*} is *N*_*g*_, i.e. $N = \sum _{g=1}^{G} N_{g}$. Assume that the feature ${f_{i}^{g}}$ is the *i*^*th*^ feature in group *g*, where its weight is ${w_{i}^{g}}$. Assume that each example *m* is associated with a label *y*_*m*_ indicating the label of the example. A visual representation for the data is depicted in Fig. 1.

**Fig. 2:**
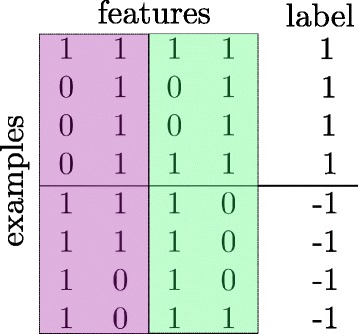
Toy example. Four samples from each class with 4 features in 2 groups. Each group has two features. Features 2 and 4 are jointly the discriminative features although feature 1 is the discriminative feature for group 1 and feature 4 is the discriminative feature for group 2, separately

The naïve approach to extract one representative feature from each group is to look at each group separately and extract the feature that maximizes the class separation (or minimizes loss function) within the observed group. However, with this approach the possible interactions among features from different groups are not considered. For example, the data in Fig. 2 (we show binary data for better visualization) show that selecting the best feature from each group separately does not guarantee a global solution. If we applied any feature selection method on group 1 and group 2 *separately* to find the best informative features, then the first feature from group 1 and second feature from group 2 will be selected because they are more discriminative than the other features. However, these two features (feature 1 and 4) combined will have one misclassification example because the first and last examples have exactly the same features set but from different classes. On the other hand, if we look at both groups *simultaneously* then we can see that features 2 and 4 are the most discriminative features where class 1 is predicted when both features are 1, and class -1 is predicted otherwise. We emphasize that the objective is not to learn a classification model, but instead we select representative features that could be used later to learn a classification model.

We propose a method that simultaneously finds a representative feature from each group. Let us assume that $\boldsymbol f^{g} = ({f_{1}^{g}} {f_{2}^{g}} \ldots f_{N_{g}}^{g})^{T}$ is a column vector representing all features for the group *g* (Fig. 1), $\boldsymbol {w}^{g}=({w_{1}^{g}} {w_{2}^{g}} \ldots w_{N_{g}}^{g})^{T}$ is a column weight vector for all features in the group *g*, and ***w***=(***w***^1^***w***^2^…***w***^*G*^). If we do not impose any restrictions on weights ***w***, the optimal weights can be found by minimizing a loss function *ℓ* over *M* examples in the training data *D*. The objective becomes minimization of the loss function with respect to the weights ***w***.

(1) $$ \underset{\boldsymbol{w}}{\text{minimize}} \quad \mathcal{L}_{1} = \sum\limits_{m=1}^{M}\ell (D)  $$

where *ℓ*(*D*) is the loss induced from the dataset *D*. We can use any loss function as long as the function is convex to ensure a global solution. In order to show that our formulation can incorporate different loss functions, we utilized the class separation loss and the logistic loss in experiments on gene expression and synthetic data, respectively; see Additional file 1 for details.

To extract one feature from each group we need to have constraints on ***w***. Therefore, we solve the following constrained optimization problem

(2a) $$\begin{array}{*{20}l} & \underset{\boldsymbol{w}}{\text{minimize}} \quad \mathcal{L}_{1}  \end{array} $$

(2b) $$\begin{array}{*{20}l} &\text{subject to} \quad {w_{i}^{g}} \in\{0,1\}, \quad \forall g\in\{1,2,\ldots,G\},i\in\{1,2,\ldots,N_{g}\},  \end{array} $$

(2c) $$\begin{array}{*{20}l} & \quad\quad\quad\,\, \sum\limits_{i=1}^{N_{g}} {w_{i}^{g}} =1, \quad\quad \forall g\in\{1,2,\ldots,G\},  \end{array} $$

The constraint (2b) ensures that the weights are binary (either the feature is selected or not), while the constraint (2c) ensures that the sum of the weights within the observed group is 1. These two constraints combined ensure that only one feature from each group is selected. The problem is combinatorial optimization with binary constraints, which is hard to solve. Our goal is to relax these constraints. By relaxing ${w_{i}^{g}}$ to be within the range [0,1], we obtain the following optimization function:

(3a) $$\begin{array}{*{20}l} & \underset{\boldsymbol{w}}{\text{minimize}} & & \mathcal{L}_{1} \end{array} $$

(3b) $$\begin{array}{*{20}l} &\text{subject to} & & {w_{i}^{g}} \geq 0, & \forall i,g, \end{array} $$

(3c) $$\begin{array}{*{20}l} & & & \sum\limits_{i=1}^{N_{g}} {w_{i}^{g}} =1, & \forall g,  \end{array} $$

(3d) $$\begin{array}{*{20}l} & & &\max_{i=1 \ldots N_{g}}{w_{i}^{g}} = 1,& \forall g.  \end{array} $$

The constraint (3d) ensures that the maximum weight within each group is 1. Therefore, constraints (3c) and (3d) jointly ensure that all weights within each group are 0 except only one weight that has value 1, which means that we select one feature from each group. However, all these prototypes are selected simultaneously such that the joint effects among them are considered.

**Fig. 3:**
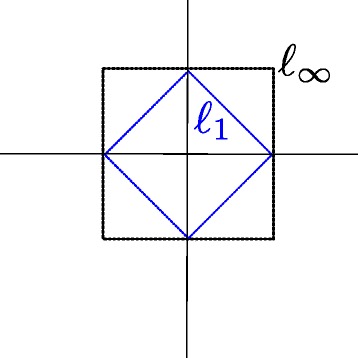
*ℓ*_1_ and *ℓ*_*∞*_ norms. *ℓ*_*∞*_ is an upper bound for *ℓ*_1_ norm

***Note.*** In case of positive weights, the constraint (3c) can be considered as *ℓ*_1_ norm, whereas the constraint (3d) is the *ℓ*_*∞*_ norm. Since *ℓ*_*∞*_ norm is an upper bound for *ℓ*_1_ norm as illustrated in Fig. 3 it might appear that there is a redundancy between these two norms. However the set of weights returned by the intersection of the two norms is different from the set of weights returned by either norm solely [21]. In particular, the intersection of the two norms forces all weights to be zero except one weight of a positive value *c* (in our formulation *c*=1 for simplicity). Therefore, using these two norms is essential in order to choose only one representative feature form each group.

If we solve the optimization problem (3a) then we achieve what we want in order to select one feature from each group simultaneously. However, the problem (3a) has equality constraints, which is not easy to solve. Therefore, we relax equality constraints by introducing penalized terms in the objective function in order to obtain its Lagrange formulation:

(4a) $$\begin{array}{*{20}l} &\underset{\boldsymbol{w}}{\text{minimize}} \quad \mathcal{L}_{1} + \lambda_{1}\! \sum\limits_{g=1}^{G}\!\left(\!\sum\limits_{i=1}^{N_{g}} {w_{i}^{g}} -1\!\right)^{2} + \lambda_{2}\! \sum\limits_{g=1}^{G}\!\left(\!\!1 - \max_{i=1 \ldots N_{g}}{w_{i}^{g}}\!\!\right)  \end{array} $$

(4b) $$\begin{array}{*{20}l} &\text{subject to} \quad {w_{i}^{g}} \geq 0 \qquad\qquad\qquad\qquad\qquad\qquad\qquad\!\!\!\! \forall i,g.  \end{array} $$

where *λ*_1_>0 and *λ*_2_>0 are the Lagrangian multipliers. The first penalization term is the difference between the sum of weights and 1. Since the sum of weights can be larger or smaller than 1 and, hence, the difference can be positive or negative; therefore, we instead penalize the quadratic term. The second penalization term is to penalize the difference between the maximum and 1, which can not be negative because the maximum can not be larger than 1 according to constraint (4b). Higher value of *λ*_2_ forces the weight of the representative feature to reach the maximum and, therefore, validates the equality constraint (3d). Since the main objective is to force one of the weights to be large (not necessarily reaching the maximum) and the remaining weights to be very close to zero, the value of *λ*_2_ is not set to be very high (similarly *λ*_1_). As explained in Additional file 1, values of these two parameters are set to *λ*_1_=*λ*_2_=100 to balance the two constraints.

The optimization problem (3a) is not easy to solve because the *max* function is not differentiable. Therefore, we approximate it with convex differentiable *log*-*sum*-*exp* function [22]. We start with the following lower bound for the *max* function

(5) $$\begin{array}{*{20}l} \max_{i=1..N_{g}} {w_{i}^{g}} &\geq \log\left(\sum\limits_{i=1}^{N_{g}} e^{{w_{i}^{g}}}\right)-\log{N_{g}}  \\ 1-\max_{i=1..N_{g}} {w_{i}^{g}} &\leq 1-\log\left(\sum\limits_{i=1}^{N_{g}} e^{{w_{i}^{g}}}\right)+\log{N_{g}}. \end{array} $$

which means that the second penalization term is upper bounded with a smooth function. Let us define

$$\begin{array}{*{20}l} \mathcal{L}_{2} & = \sum\limits_{g=1}^{G}\left(\sum\limits_{i=1}^{N_{g}} {w_{i}^{g}} -1\right)^{2} \\ \mathcal{L}_{3} & = (-1) \sum\limits_{g=1}^{G} \left(1-\log\left(\sum\limits_{i=1}^{N_{g}} e^{{w_{i}^{g}}}\right)+\log{N_{g}}\right). \end{array} $$

Then, we combine (5) and (4) to get the following optimization problem:

(6) $$  \begin{aligned} &\underset{\boldsymbol{w}}{\text{minimize}} & & \mathcal{L}_{1} + \lambda_{1} \mathcal{L}_{2} - \lambda_{2} \mathcal{L}_{3} \\ &\text{subject to} & & {w_{i}^{g}} \geq 0 & \forall i,g. \end{aligned}  $$

$\mathcal {L}_{1}$ is a convex loss function, $\mathcal {L}_{2}$ is a quadratic function and therefore convex, and $\mathcal {L}_{3}$ is convex because *log*-*sum*-*exp* is a convex function. Then, the objective function (6) becomes difference of two convex functions. In order to solve this problem we have applied a recent convex-concave procedure (CCCP) [23, 24]. CCCP linearizes the concave function around a solution obtained in the current iterate with tangent hyperplane function, which serves as an upper-bound for the concave function. This leads to a sequence of convex programs where the convergence of the method is guaranteed [25].

Therefore, in each iteration we solve the convex optimization problem:

(7) $$  \begin{aligned} &\underset{\boldsymbol{w}}{\text{minimize}} && \mathcal{J}=\mathcal{L}_{1} + \lambda_{1} \mathcal{L}_{2} - \lambda_{2} \boldsymbol{w} \cdot \left(\frac{d\mathcal{L}_{3}}{d\boldsymbol{w}}\right)_{\boldsymbol{w}=\boldsymbol{w}^{t}} \\ &\text{subject to} && {w_{i}^{g}} \geq 0, \quad\quad\quad\quad \forall i,g. \end{aligned}  $$

where the term $(d\mathcal {L}_{3}/d\boldsymbol {w})_{\boldsymbol {w}=\boldsymbol {w}^{t}}$ is the derivative of $\mathcal {L}_{3}$ at the current iterate ***w***^*t*^.



The application of CCCP is shown in Algorithm 1. The advantage of CCCP is that no additional hyper-parameters are needed. Furthermore, each update is a convex minimization problem and can be solved using classical and efficient convex apparatus. Since we now have a smooth, differentiable objective function $\mathcal {J}$ with only inequality constraints, we can use any optimization algorithm for solving the problem. In order to solve the problem efficiently we compute first derivatives of the objective function with respect to the weights ***w***, and approximate the Hessian with a diagonal matrix. In Additional file 1, we show the derivation of Jacobian and Hessian matrices for the logistic loss [26] and class-separable loss functions [19].

The trust-region-reflective algorithm [27] is the fastest optimization algorithm for solving (7). However, in our application it is not efficient for large scale problems. In the next section, we develop a customized optimization algorithm based on coordinate descent that is four times faster than standard apparatus.

### Block coordinate gradient descent

Coordinate gradient descent is a simple technique that is surprisingly efficient and scalable [28]. In general, given convex and differentiable function, the coordinate descent algorithm minimizes the function along each coordinate axis ${w_{i}^{g}}$, nevertheless, it is guaranteed that the algorithm will converge to the global optimal solution [29]. Moreover, in many cases we can replace individual coordinates with blocks of coordinates, e.g. coordinates ***w***^*g*^ for a group *g* [30].

In order to develop a block coordinate gradient descent (BCGD) algorithm to solve (7), we build our work on the seminal work of [31, 32], where they have developed an algorithm to solve a smooth function with bound constraints as in (7). The key idea of the algorithm is to iteratively combine a quadratic approximation of the objective function $\mathcal {J}$ at ***w*** to generate a feasible direction ***d*** with an additional line search to find the best move along that direction. The procedure continues in iterative mode until convergence. Precisely, BCGD Algorithm (2) iteratively runs over four steps until convergence. In the first step, the algorithm identifies a set of features (coordinates) in order to optimize (the iterations are performed such that each *T* consecutive iterations run over the entire ***w***). Typically, the algorithm iterates over those non-zero weights (active set) and optimize their corresponding features. In the second step, the algorithm approximates the objective function as a quadratic optimization at the active set and then performs line search in step 3 to find the best step size to move along the direction of the quadratic approximation. This ensure a feasible movements towards the minimum. Finally, it updates only the active set weights. The key issue of Algorithm (2) is how to identify the active set so that the algorithm runs efficiently and optimizes the active weights.



#### Active weights for solving (7)

Cyclically updating one coordinate at a time in coordinate descent process might slow down the optimization for large scale problems. Therefore, in our approach the update is performed based on blocks of coordinate [30]. Our application is easily fitted in this situation where the blocks can be naturally chosen based on groups of features. In each iteration we update the weights of all features within one group.

Nevertheless, in our application we did not see benefit from iterating over each group. Instead, we initially set the active set as the entire ***w***, and we update the parameters based on all coordinates at once. Such an update is successfully used in a previous study where it is noted that “*after a complete cycle through all the variables, we iterate on only the active set till convergence*” [28]. Then, after a few iterations some of the groups get stable (i.e., one of the features becomes close to 1 and the rest become close to 0). In this case, we do not need to optimize this group anymore and we can exclude that group from the active set. If we keep that group in the working set the algorithm will try to fit precisely the weights of the features within that group while the selected feature will remain stable. In other words, the optimizer will try to move the max weight to be closer to 1 while keeping the rest of the weights closer to 0. Therefore including those groups in the working set will just slow down the optimization without changing the representative for those groups. This resembles the well know active-set method, which iteratively predicts a correct split of zero and non-zero elements in ***w*** and optimizes the function based on only non-zero weights [33].

## Results and discussion

### Microarray gene expression

We compared the proposed feature selection formulation using block coordinate gradient descent (BCGD) algorithm to two baseline and two state-of-the-art feature selection filter-type methods. (1) The Pearson Correlation (PC) method which ranks the correlation between the feature and the target and selects the top *m* features; (2) Relief which is one of the most successful strategies in feature selection [34, 35]. It chooses instances randomly and changes the weights of the feature relevance based on the nearest neighbor so that it gives more weights to features that discriminate the instance from neighbors of different classes; (3) mRMR ranks the features according to the minimal-redundancy-maximal-relevance criteria [17, 36], which is based on mutual information; (4) STBIP formulates the feature selection problem as a quadratic objective function to select *m* features with maximal discriminative power and minimal redundancy [19], where the redundancy among features is computed based on Pearson correlation. We note that all methods we compare to, including our method, are filter-type feature selection methods, where the objective is to rank or select features without learning a classifier.

In order to apply our methods, we need to cluster the genes. The genes can be clustered in different ways. For example, each cluster may include genes that encode for similar polypeptides or proteins, which are often located few thousands pairs apart from each other. Gene Ontology (GO) has been utilized to cluster genes based on their common function where they are not constrained by gene expression or other properties [37]. Another way to cluster genes is to group co-expressed genes in the same cluster, which do not necessarily have similar functions [38]. For a survey on clustering genes, the reader is referred to [39, 40] and references therein. Our method is decoupled from the clustering step. However, in order to have a fair comparison with other baseline methods and to select the top *m* features selected by our method, we clustered the genes based on Pearson correlation into *m* clusters and applied our method to select one gene from each group.

The selected genes is then fed to linear SVM as the classification model. We used linear SVM because it has been shown to be effective in gene expression classification problems [9]. We evaluated the feature selection methods on five benchmark gene expression datasets [41] described in Table 1.

**Table 1:** Gene expression dataset description

Dataset	# Genes	# Samples	# Training samples
		(tumor/normal)	(tumor/normal)
Tumor14 [48]	15,009	308(90/218)	(20/20)
Lung [48]	15,009	27(20/7)	(5/5)
Myeloma [49]	12,625	173(137/36)	(20/20)
DLBCL [50]	5,469	77(58/19)	(15/15)
Colon [51]	2,000	62(40/22)	(20/20)

For each dataset, we sampled training data from each class (as indicated in the last column in Table 1) for training the feature selection method, and the remaining samples were used as test data. Using only the selected features, linear SVM was optimized on the training data using the LIBLINEAR package [42], where the parameter *C*={10^−3^,10^−2^,…,10^3^} was chosen based on a nested 3-cross validation on the training set. Note that the test data were never used for training in either the feature selection method or SVM. Since the microarray datasets are imbalanced, we used the area under the ROC curve (AUC) as the evaluation performance. The average AUC is computed based on 40 repetitions of random splits for training and test data.

**Fig. 4:**
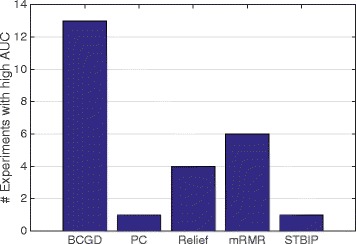
Winners. The number of experiments where each feature selection method has the best average AUC

We evaluated each method using the top *m*={20,50,100,200,1000} genes over 40 runs and computed the average AUC for each experiment. Out of 25 experiments (5 different values for *m* and 5 different microarray datasets), the number of experiments where each method has the highest average AUC is shown in Fig. 4. The proposed BCGD method has the best AUC in 13 experiments, whereas all other methods have the highest AUC in no more than 6 experiments. The results show that the proposed method has selected more accurate features than other state-of-the-art methods.

The details of these results are shown in Table 2. Each row in the table shows the average and standard deviation of AUC performance of all methods on one dataset over 40 runs for different *m* features. The last row shows the average AUC performance for each method on *all* datasets. The proposed method has on average the best AUC among all other feature selection methods, which indicates that the proposed method has selected the most discriminative features. In addition, it has the smallest standard deviation. While the standard deviation of all methods overlap but when we applied t-test on the results we found that the average AUC of the proposed method is statistically more significant than other methods in 12 out of 20 cases (*α*=0.05).

**Table 2:** Evaluation of gene selection methods on 5 benchmark datasets using the top *m* genes

	Method	*m*=20	*m*=50	*m*=100	*m*=200	*m*=1000
Tumor14	BCGD	**0.786 ±0.036**	0.797 ±0.049	**0.821 ±0.041**	**0.825 ±0.036**	**0.846 ±0.042**
	PC	0.766 ±0.047	0.786 ±0.041	0.793 ±0.041	0.805 ±0.045	0.830 ±0.033
	Relief	0.748 ±0.069	0.788 ±0.050	0.803 ±0.033	0.822 ±0.036	0.844 ±0.038
	mRMR	0.785 ±0.041	**0.803 ±0.036**	0.813 ±0.038	0.817 ±0.039	0.824 ±0.033
	STBIP	0.672 ±0.054	0.733 ±0.035	0.761 ±0.044	0.795 ±0.038	0.837 ±0.047
Lung	BCGD	**0.762 ±0.180**	**0.806 ±0.168**	**0.789 ±0.183**	**0.789 ±0.163**	0.785 ±0.169
	PC	0.732 ±0.200	0.777 ±0.189	0.756 ±0.185	0.777 ±0.188	0.789 ±0.153
	Relief	0.652 ±0.243	0.689 ±0.245	0.732 ±0.206	0.752 ±0.224	**0.797 ±0.147**
	mRMR	0.721 ±0.207	0.750 ±0.195	0.755 ±0.195	0.788 ±0.184	0.783 ±0.156
	STBIP	0.637 ±0.212	0.721 ±0.198	0.739 ±0.192	0.767 ±0.170	0.771 ±0.165
Myeloma	BCGD	0.662 ±0.077	**0.706 ±0.061**	**0.712 ±0.062**	0.709 ±0.058	0.717 ±0.061
	PC	**0.675 ±0.071**	0.694 ±0.068	0.705 ±0.058	0.709 ±0.055	0.713 ±0.056
	Relief	0.583 ±0.085	0.624 ±0.085	0.650 ±0.075	0.679 ±0.064	0.709 ±0.058
	mRMR	0.647 ±0.077	0.691 ±0.058	0.702 ±0.061	**0.715 ±0.061**	**0.721 ±0.057**
	STBIP	0.565 ±0.093	0.619 ±0.079	0.648 ±0.082	0.672 ±0.079	0.701 ±0.062
DLBCL	BCGD	**0.970 ±0.031**	0.971 ±0.034	0.975 ±0.024	0.981 ±0.023	0.987 ±0.017
	PC	0.947 ±0.041	0.957 ±0.042	0.963 ±0.047	0.964 ±0.043	0.980 ±0.027
	Relief	0.947 ±0.062	0.974 ±0.027	0.983 ±0.020	**0.988 ±0.016**	**0.990 ±0.013**
	mRMR	0.962 ±0.055	**0.981 ±0.025**	**0.987 ±0.019**	0.985 ±0.021	0.980 ±0.025
	STBIP	0.808 ±0.101	0.905 ±0.060	0.925 ±0.063	0.943 ±0.066	0.978 ±0.029
Colon	BCGD	0.874 ±0.110	0.878 ±0.101	**0.879 ±0.095**	**0.886 ±0.096**	0.858 ±0.119
	PC	0.886 ±0.164	0.879 ±0.150	0.863 ±0.150	0.868 ±0.125	0.856 ±0.115
	Relief	**0.896 ±0.113**	0.888 ±0.098	0.877 ±0.104	0.859 ±0.128	0.859 ±0.113
	mRMR	0.874 ±0.115	**0.889 ±0.104**	0.872 ±0.120	0.870 ±0.093	0.850 ±0.118
	STBIP	0.781 ±0.181	0.821 ±0.143	0.847 ±0.128	0.847 ±0.139	**0.862 ±0.115**
Average	BCGD	**0.811 ±0.148**	**0.832 ±0.132**	**0.835 ±0.133**	**0.838 ±0.127**	0.840 ±0.137
	PC	0.801 ±0.158	0.819 ±0.146	0.816 ±0.144	0.825 ±0.137	0.834 ±0.131
	Relief	0.765 ±0.191	0.792 ±0.179	0.809 ±0.159	0.820 ±0.158	**0.841 ±0.132**
	mRMR	0.798 ±0.160	0.823 ±0.145	0.826 ±0.145	0.835 ±0.133	0.832 ±0.131
	STBIP	0.693 ±0.167	0.760 ±0.153	0.784 ±0.148	0.805 ±0.141	0.830 ±0.138

The average and standard deviation of AUC is reported for each experiment over 40 runs. Last raw shows the average AUC over all datasets. Bold represents the best AUC

**Gene-GO enrichment analysis.** We have performed an enrichment analysis to find which gene ontology (GO) terms are over-represented using annotations for the selected genes. Therefore, in order to perform a function annotation analysis, the selected 100 BCGD genes from the Myloma dataset were submitted to DAVID Bioinformatics Resources [43, 44]. The top 10 GO terms are reported in Table 3. The last column in the table is the modified Fisher exact *p*-value, which is the probability of seeing at least *x* genes out of *n* genes in the list annotated to a particular GO term, given the proportion of genes in the whole genome that are annotated to that GO Term.

**Table 3:** Top 10 GO terms enriched in the BCGD selected genes from the Myleoma dataset

GO ID	Ontology	GO term	Percentage	*P*-value
GO:0044444	Cellular Component	cytoplasmic part	45.8	2.0E-4
GO:0015629	Cellular Component	actin cytoskeleton	8.3	6.8E-4
GO:0044449	Cellular Component	contractile fiber part	5.2	3.4E-3
GO:0043412	Biological Process	biopolymer modification	19.8	4.3E-3
GO:0032991	Cellular Component	macromolecular complex	30.2	4.3E-3
GO:0043292	Cellular Component	contractile fiber	5.2	4.4E-3
GO:0005622	Cellular Component	intracellular	75.0	6.1E-3
GO:0005515	Molecular Function	protein binding	60.4	6.2E-3
GO:0005737	Cellular Component	cytoplasm	55.2	6.7E-3
GO:0008081	Molecular Function	phosphoric ester hydrolase activity	7.3	1.1E-2

Percentage is the percentage of BCGD genes involved in the corresponding term

Myeloma is a cancer of plasma cells in which abnormal plasma cells multiply uncontrollably in the bone marrow and occasionally in other parts of the body. We can see from Table 3 that *cytoplasmic part* and *cytoplasm* terms are enrighed by the BCGD selected genes. Also, actin cytoskeleton is a mediator of apoptosis, which leads to cancer [45]. Furthermore, we have performed a disease association analysis using WebGestalt [46, 47]. Table 4 shows the list of top 10 enriched diseases and the number of genes in the gene list for the disease. It is shown that 9 of those diseases are directly connected to cancer.

**Table 4:** Top 10 diseases associated with the BCGD selected genes

Disease	# Gene	*P*-value
Stress	8	1.6E-3
Nevi and Melanomas	6	1.6E-3
Large granular lymphocytic leukemia	3	1.7E-3
cancer or viral infections	10	2.1E-3
Hemoglobinuria	3	2.1E-3
Neuroendocrine Tumors	5	2.1E-3
HIV	9	2.1E-3
Leukemia, T-Cell	5	2.1E-3
Corneal Neovascularization	3	2.1E-3
Leukemia	7	2.1E-3

The number of genes in the selected BCGD gene list associated with the disease. *P*-value is adjusted by the multiple test adjustment

**Fig. 5:**
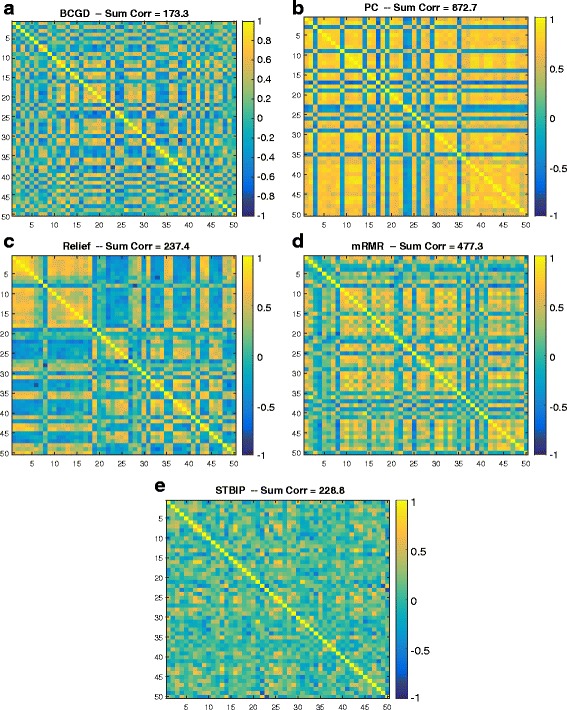
Similarity among selected features. The similarity matrix between all genes selected by 5 feature selection methods

**Diversity of genes.** In order to show the diversity of the selected genes by each feature selection method, we consider the DLBCL microarray dataset as a case study. We plot the similarity (Pearson correlation) matrix between all selected genes by each method and computed the sum of the similarity matrix. The lower the value the less similar the features are. Figure 5 shows the similarity matrix for each method. PC has the most redundant features among all methods, where the sum of its similarity matrix (872.7) is significantly greater than any other method. This is consistent with the fact that the method ranks the genes based solely on their correlation to the target regardless of the similarity among the selected genes, and hence the selected genes are very similar to each other, which might reduce the interpretability of the model. On the other hand, the proposed BCGD method has the lowest similarity value (173.3) indicating that the method has selected the most diverse (less redundant) genes while being accurate. The STBIP was the second successful method to choose the less redundant features.

### Efficiency of BCGD on synthetic data

The proposed feature selection formulation can be solved using any standard optimization algorithm. We have used trust region reflective (TR) algorithm as it is the best and fastest implemented algorithm in Matlab for the proposed constrained optimization problem (7). However, we have developed an efficient block coordinate gradient descent (BCGD) algorithm that is four times faster than the standard algorithm for high dimensional applications (million of features). In order to show the efficiency of BCGD, we have conducted several synthetic experiments, where all synthetic datasets have been generated using the process described in Additional file 1. First, we conducted experiments to show the efficacy of utilization of active set and how that contributed to the reduction in computational cost of the BCGD algorithm. Then, we compared the computational time of BCGD and TR in 42 settings with different number of features *N*={10*e*^3^,100*e*^3^,200*e*^3^,400*e*^3^,600*e*^3^,800*e*^3^,1*e*^6^} distributed over different number of groups *G*={100,200,400,600,800,1000}.

#### Utilization of active set

The BCGD algorithm does not update the weights for all groups at each iteration. Instead, it updates the entire weight vector (i.e., all groups) at the first few iterations and then at each next iteration it identifies the non-stable groups and optimizes only those groups. We hypothesize that if the group has clear discriminative features then the observed group will become stable at earlier iterations, while groups that have most confusing features will go for longer iterations.

**Fig. 6:**
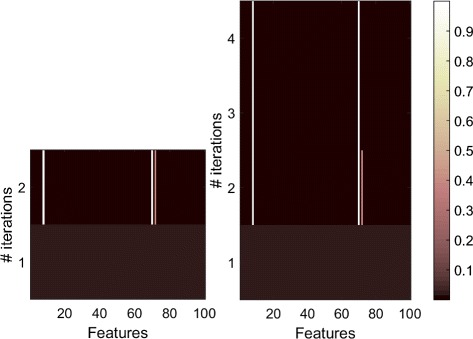
Active set. The *right panel* shows the BCGD algorithm when updating the entire set of weights at each single iteration. The *left panel* shows the BCGD algorithm when using the active set groups. As soon as the group becomes stable, BCGD does not optimize that group, which results in reduction in computational time

We compared the BCGD algorithm when using the active set (optimizing only the non-stable groups) and without using the active set (optimizing the entire ***w*** in each iteration). For easier visualization, the simulation was done on 100 features with 2 groups, where the ground truth features are 8 and 70. The left panel of Fig. 6 shows BCGD when using the active set (optimizing only the non-stable groups), while the right panel shows the algorithm optimizing the entire ***w*** in each iteration. It clear from the figure that the algorithm was able to find the correct features after 2 iterations (as depicted in the left panel). However, feature 72 had value greater than zero (∼0.2) and feature 70 had value close to 1 (∼0.8). The BCGD algorithm using active set (left panel) stops at this iteration and considered group 2 as stable. If we let the algorithm continue and optimize the entire ***w*** (right panel), then it needs two more iterations to find the optimal weights. However, the selected features are still the same and the only change is that the weight of feature 70 will be fully optimized (very close to 1) and the weight of the rest of features will be 0. Therefore, we benefit from this observation and utilize the active set in order to reduce the computational cost of the BCGD algorithm.

**Fig. 7:**
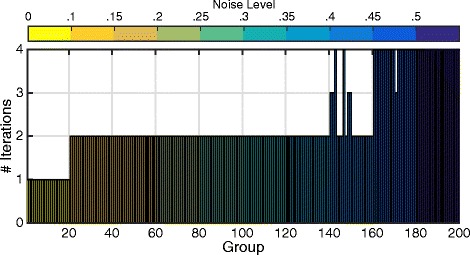
Groups with different noise levels. X-axis represents the groups where each consective 20 groups have the same noise level represented in the corresponding colorbar at the top of the figure. Y-axis represents the number of iterations needed by BCGD algorithm to optimize the weights of the corresponging group. Easy groups (less noisy) are terminated early while difficult groups (more noisy) are terminated late

In order to further simulate this situation in a large scale, we created a dataset with 20k features distributed evenly over 200 groups and added different noise levels to different groups. Precisely, we added 0 noise to the representative features of each of the first 20 groups. Then, we added 10 % noise to each group in the next 20 groups, which means that 10 % of samples in the representative features in each group are flipped. Then, we added 15 % noise in the next 20 groups, and so on until we add 50 % of noise in the last 20 groups. The result of applying BCGD is shown in Fig. 7. The figure shows the number of iterations needed by the BCGD algorithm to optimize the weights for each group. It is clear that the first 20 groups (with 0 noise) became stable after only 1 iteration. That means that BCGD does not update the weights of these 20 groups afterwards, which contributes to the reduction in computational time of BCGD. On the other hand, the last 20 groups (with higher noise) lasted for all iterations, because it was not easy for the algorithm to identify the representative features from these groups.

#### Scalability

To show the efficiency of the proposed BCGD algorithm, experiments were conducted to compare the running time for both algorithms TR and BCGD. Ten datasets were generated with 100 samples with *N* features distributed over *G* groups. We varied the number of groups *G*={100,200,400,600,800,1000} and the number of features *N*={10*e*^3^,100*e*^3^,200*e*^3^,400*e*^3^,600*e*^3^,800*e*^3^,1*e*^6^} and. Then, both algorithms were applied on each dataset and the results were computed as the average over all 10 datasets.

**Fig. 8:**
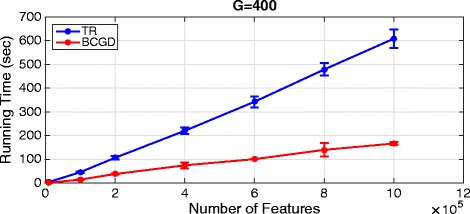
Running time. Running time for trust region (TR) and BCGD on synthetic data with varying number of features distributed over *G*=400 groups

In all settings (|*N*|×|*G*|=42), both algorithms have identified the ground truth features. However, the proposed BCGD algorithm is significantly faster than TR. Figure 8 shows the computational time comparison between both algorithms with fixed *G*=400 and varying number of features. When the number of features increases the running time of both algorithms increases. However, the speedup of BCGD over TR increases as the number of features increases indicating the applicability of BCGD on high dimensional data than just using standard optimization algorithm.

**Fig. 9:**
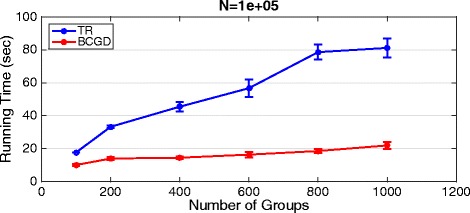
Running time. Running time for trust region (TR) and BCGD on synthetic data with *N*=100*e* ^3^ features distribted over different number of groups

Figure 9 shows the comparison with fixed number of features *N*=100*e*^3^ and varying number of groups. Again, when the number of groups increases the running time of both algorithms increases. This is intuitive because the objective function is to optimize the weights in each group, therefore, increasing the number of groups would increase the running time of the optimization algorithms as shown in the figure. Moreover, BCGD is faster than TR in all cases. The full details of the computational time comparison results are shown in the Additional file 1.

## Conclusion

Feature selection method was proposed to select features in order to jointly maximizing the discriminative power of the features. This is performed by considering the structural relationships among features, where the features are grouped based on prior knowledge. The feature selection problem is then formulated as selecting a feature from each group. We developed a block coordinate gradient descent algorithm to solve the optimization function. The results of comparing the proposed method with 4 stat-of-the-art methods on five bench mark gene expression datasets showed evidence that the proposed method was accurate on 13/25 experiments where the other methods was accurate in no more than 6/25 experiments. In addition, several synthetic experiments were conducted to show the efficiency of the proposed BCGD algorithm over the standard optimization algorithm. The BCGD algorithm was four times faster than the standard algorithms indicating the applicability of BCGD on high dimensional data. In future work, we will investigate convergence properties for the proposed method. In addition, it might be interesting to learn the clusters of genes simultaneously with the feature selection method.

## Additional file

Additional file 1 **Supplementary materials.** The supplementary PDF file contains relevant information omitted from the main manuscript such as: (1) other applications for the proposed features selection method; (2) derivation for two loss functions used in the experiments; (3) implementation details for BCGD; (4) synthetic data generation process; and (5) scalability results that are not reported in the main manuscript. (PDF 271 KB)
