# Supplementary material for: Structured feature selection using coordinate descent optimization
Source: BMC Bioinformatics. 2016 Apr 8;17:158. doi: 10.1186/s12859-016-0954-4 (PMC4826549; doi:10.1186/s12859-016-0954-4)
Supplement: Supplementary file 1 — Supplementary materials. The supplementary PDF file contains relevant information omitted from the main manuscript such as: (1) other applications for the proposed features selection method; (2) derivation for two loss functions used in the experiments; (3) implementation details for BCGD; (4) synthetic data generation process; and (5) scalability results that are not reported in the main manuscript. (PDF 271 KB) [file 12859_2016_954_MOESM1_ESM.pdf]

Supplementary Materials for *Fast Structured Feature Selection using Coordinate Descent Optimization*

Applications for the Proposed Feature Selection

Multivariate Time Series Classification

The objective of multivariate time series classification is to assign a class label to multivariate time series [?], e.g. gene expression measurements over time for each patient, where each patient is classified as control or having the condition [?]. This problem can be addressed by using discriminative multivariate temporal patterns that are extracted from each class [1, 2]. The ideal patterns efficiently discriminate among patients from different classes and therefore could be used as interpretable way to predict whether the patient is developing condition [?]. One example of such interpretable multivariate pattern is that if gene X and gene Y are up-regulated at the same time followed by down-regulation of gene Z then the patient is developing the condition. In order to extract such patterns, one can extract all patterns from gene X as one group and all patterns extracted from gene Y as another group, and so on. In other words, the grouping structure among genes is based on all patterns extracted from one variable (gene). The matrix in Figure 1 (in the paper) can be constructed such that each entry is 1 if the corresponding pattern (column) is present in the time series of the patient (row), and 0 otherwise [?]. Hence, the discriminative multivariate pattern can be extracted such that at most one univariate pattern is extracted from each group (gene), e.g. at most one pattern from gene X, one pattern form gene Y, etc. Our feature selection method can be easily applied to extract exactly one pattern from each group. Then, after extracting exactly one pattern form each group, another feature selection method [?] can be applied to reduce the dimensionality of the extracted multivariate pattern.

Analytics in Sports

One major objective of analytics in sports is to enhance team performance by selecting the best possible players and make the best possible decisions on the field or court [?]. Imagine that a coach needs to select a set of best players for the team. Intuitively, the set of all possible players can be grouped (based on their positions in the field) into G groups where each group contains all players who play in that position. Since the objective is to select the best team one may claim that the problem can be solved by selecting the best player in each position separately. However, using that approach the synergism among the players are not considered. For example, players 1 and 2 are the best players for positions A and B, respectively, but the players might not be cooperative. So, including players 1 and 2 in the same team might reduce the performance of the entire team. Therefore, the idea is to select one player from each group such that the selected team has the best performance.

Derivation of Loss Functions

Logistics Loss

Assume that the logistic function is the loss function of interest [3, ?], then:

$$\mathcal{L}_1 = \sum_{m=1}^{M} \log\left(1 + e^{-y_m \sum_{g=1}^{G} \sum_{i=1}^{N_g} w_i^g f_{im}^g}\right) \tag{1}$$

where y_m is the label for the mth example, and f_{im}^g is the ith feature in the group g for the mth example. Assume that

$$\hat{y}_m = \sum_{g=1}^{G} \sum_{i=1}^{N_g} w_i^g f_{im}^g$$

Then, the Jacobin and Hessian matrix are defined as:

$$\frac{\partial \mathcal{J}}{\partial w_i^g} = \sum_{m=1}^{M} \frac{y_m^2 \hat{y}_m f_{im}^g}{1 - y_m \hat{y}_m} + 2\lambda_1 (\sum_{i=1}^{N_g} w_i^g - 1) - \lambda_2 \left(\frac{e^{w_i^g}}{\sum_{i=1}^{N_g} e^{w_i^g}} \right)_{\boldsymbol{w}=\boldsymbol{w}^t}, \tag{2}$$

$$\frac{\partial^2 \mathcal{J}}{\partial w_i^g \partial w_i^g} = \sum_{m=1}^{M} \frac{-y_m \hat{y}_m}{(1 - y_m \hat{y}_m)^2} (f_{im}^g)^2 + 2\lambda_1 \tag{3}$$

Class Separation Loss

In order to precisely define the class separation loss, let us assume that $IntraCS$ is the intra-class distances, which is the sum of the distance between all instances from one class C.

$$IntraCS = \sum_{m,k \in C} \|\boldsymbol{w} \odot \boldsymbol{f}_{:m} - \boldsymbol{w} \odot \boldsymbol{f}_{:k}\|^2 \tag{4}$$

where $f_{:m}$ is the feature vector for the mth example, $\boldsymbol{w}$ is the weight vector for all features, $\odot$ is the pairwise multiplication, and the sum runs over all examples m and k from the same class C.

Similarly, the inter-class distances $InterCS$ is defined as the sum of the distances between all examples from different classes.

$$InterCS = \sum_{m \in C_1, k \in C_2} \|\boldsymbol{w} \odot \boldsymbol{f}_{:m} - \boldsymbol{w} \odot \boldsymbol{f}_{:k}\|^2 \tag{5}$$

where the sum runs over all examples from different classes C_1 and C_2.

The objective of the class separation loss is to minimize the intra-class distances and maximizes the inter-class distances. Therefore, the loss function is defined as

$$\underset{\boldsymbol{w}}{\text{minimize}} \quad IntraCS - InterCS \tag{6}$$

Following [4], the class separation CS loss function can be defined as:

$$CS = IntraCS - InterCS \tag{7}$$

$$= \sum_{m,k \in C} \|\boldsymbol{w} \odot \boldsymbol{f}_{:m} - \boldsymbol{w} \odot \boldsymbol{f}_{:k}\|^2 - \sum_{m \in C_1, k \in C_2} \|\boldsymbol{w} \odot \boldsymbol{f}_{:m} - \boldsymbol{w} \odot \boldsymbol{f}_{:k}\|^2 \tag{8}$$

$$= \sum_{m,k} \|\boldsymbol{w} \odot \boldsymbol{f}_{:m} - \boldsymbol{w} \odot \boldsymbol{f}_{:k}\|^2 A_{mk} \tag{9}$$

where A_{mk} is 1 if the examples m and k are in the same class C, and -1 otherwise. Let us assume that L is the Laplacian of the adjacency matrix A, then equation (9) can be rewritten as linear term [4]:

$$CS = Q^T \boldsymbol{w} \tag{10}$$

where Q is a column vector such that $Q_i = diag(\boldsymbol{f}^T L \boldsymbol{f})$, Q^T is the transpose of Q, and Q_i^g is the value of the entry in Q that corresponds to w_i^g. Then, the Jacobin and Hessian matrix are defined as:

$$\frac{\partial \mathcal{J}}{\partial w_i^g} = Q_i^g + 2\lambda_1 (\sum_{i=1}^{N_g} w_i^g - 1) - \lambda_2 \left(\frac{e^{w_i^g}}{\sum_{i=1}^{N_g} e^{w_i^g}} \right)_{\boldsymbol{w}=\boldsymbol{w}^t}, \tag{11}$$

$$\frac{\partial^2 \mathcal{J}}{\partial w_i^g \partial w_i^g} = 2\lambda_1 \tag{12}$$

Implementation Details

Convergence Criteria

The convergence of the optimization algorithm (either the standard trust-region-reflective or the proposed BCGD algorithm) can be based on the norm of the step tolerance. However, we found that using the step tolerance as

the convergence test while converges to the minimum it makes more iterations than necessary in both algorithms. In other words, the algorithm identifies the representative (one feature from each group) in just few iterations and the rest of iterations is just to optimize the weights. Since we are not interested in finding the optimal weights of the features (because all of them would approach zero and the prototypes would approach 1) but instead we are interested in finding the prototypes themselves, we tested the convergence of the algorithm based on the selected features from the last 3 iterations. If the same features are selected for the last 3 iterations, we consider the algorithm converges. While this convergence test is heuristic, we found it trades the scalability for *optimality* and they gave the same set of features while the heuristic one is much faster. To have fair comparison between both trust-region-reflective and BCGD algorithms, we used the same convergence test for both algorithms.

Parameters for Gene Expression Experiments

In the gene expression experiments and in order to have a fair comparison to STBIP algorithm, the class separation loss function is used. The value of the Lagrangian parameters $\lambda_1 = \lambda_2 = 100$ in all gene expression experiments. The value of the Lagrangian parameter is chosen to balance between the two terms: minimization of the loss function and the constraints on the weights to choose a representative feature from each group. Higher value of the parameter puts more emphasize on validating the constraints, whereas lower value puts more emphasize on minimizing the loss function.

Results

Synthetic Data Generation Process

Let us assume that the number of examples is M and each class has the same number of examples $M/2$. Then, we generated N features, where each feature is generated uniformly from $\{0, 1\}$, and split the features randomly into G groups (each group might have different number of features). Then, we chose one feature from each group and replaced it by another discriminative feature that has ones in one class and zeros in the other class. The rational for this generation process is that we need to compare the computational time between trust region and block coordinate gradient descent algorithm.

Scalability of BCGD

We compared the average running time of both algorithms TR and BCGD on different number of groups as shown in Figure 1, and on different number of features as shown in Figure 2.

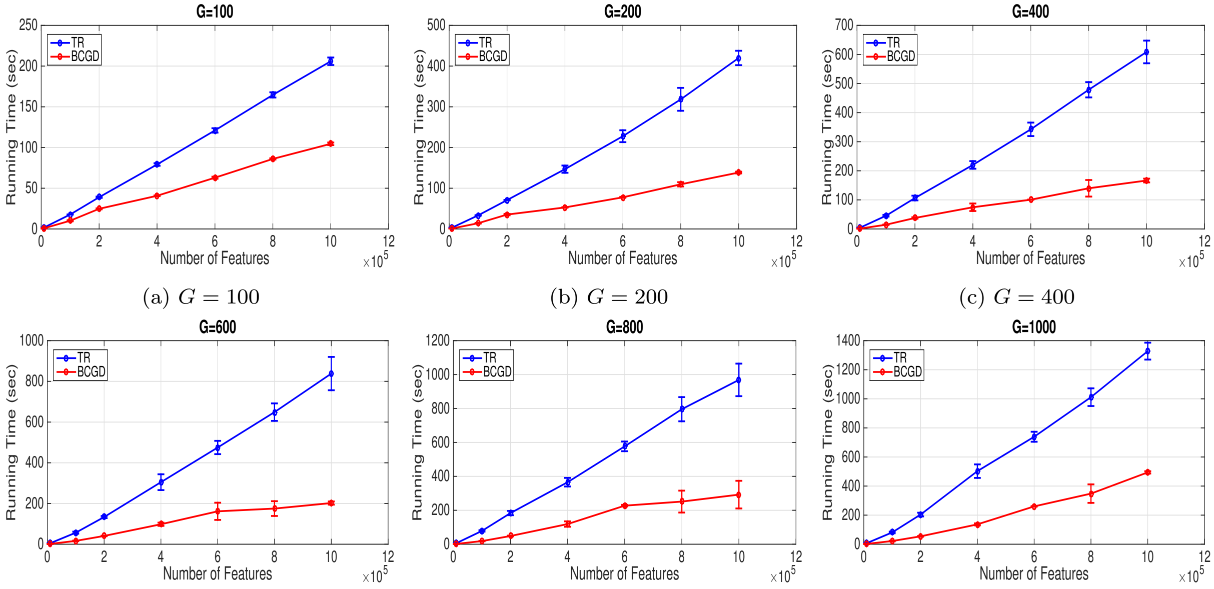

(a) $G = 100$ (b) $G = 200$ (c) $G = 400$

(d) $G = 600$ (e) $G = 800$ (f) $G = 1000$

Figure 1: Running time for both algorithms.

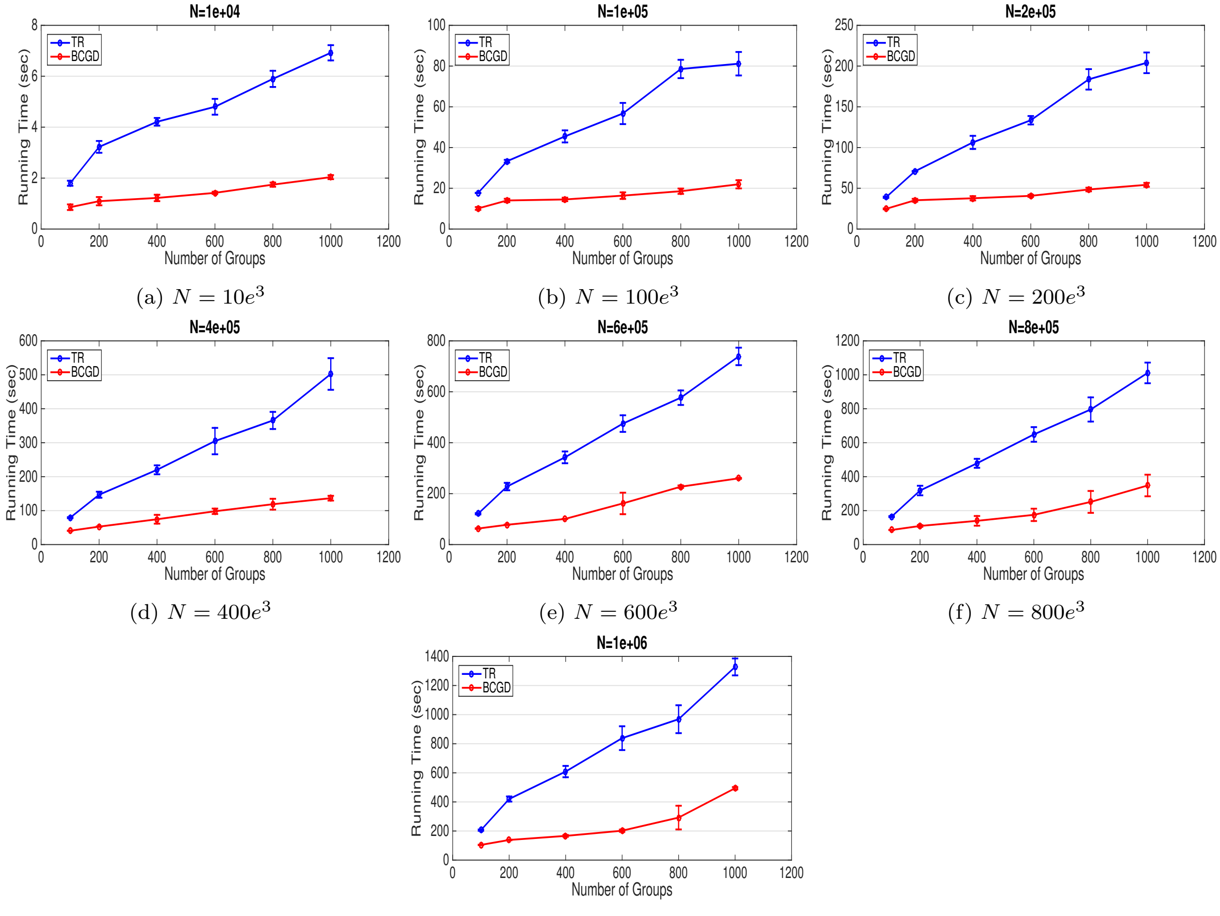

(a) $N = 10e^3$ (b) $N = 100e^3$ (c) $N = 200e^3$

(d) $N = 400e^3$ (e) $N = 600e^3$ (f) $N = 800e^3$

(g) $N = 1e^6$

Figure 2: Running time for both algorithms.

References

[1] Ghalwash, M.F., Obradovic, Z.: Early classification of multivariate temporal observations by extraction of interpretable shapelets. BMC Bioinformatics **13** (2012). doi:10.1186/1471-2105-13-195

[2] Ghalwash, M.F., Radosavljevic, V., Obradovic, Z.: Extraction of interpretable multivariate patterns for early diagnostics. In: IEEE 13th International Conference on Data Mining (ICDM), pp. 201–210 (2013). IEEE

[3] Rosasco, L., Vito, E., Caponnetto, A., Piana, M., Verri, A.: Are loss functions all the same? Neural Computation **16**(5), 1063–1076 (2004)

[4] Lan, L., Vucetic, S.: Multi-task feature selection in microarray data by binary integer programming. In: BMC Proceedings, vol. 7, p. 5 (2013). BioMed Central Ltd
